# Protocol to disseminate a hospital-site controlled intervention using audit and feedback to implement guidelines concerning inappropriate treatment of asymptomatic bacteriuria

**DOI:** 10.1186/s13012-018-0709-x

**Published:** 2018-01-19

**Authors:** Barbara W. Trautner, Pooja Prasad, Larissa Grigoryan, Sylvia J. Hysong, Jennifer R. Kramer, Suja Rajan, Nancy J. Petersen, Tracey Rosen, Dimitri M. Drekonja, Christopher Graber, Payal Patel, Paola Lichtenberger, Timothy P. Gauthier, Steve Wiseman, Makoto Jones, Anne Sales, Sarah Krein, Aanand Dinkar Naik, The Less is More Study Group, Barbara W. Trautner, Pooja Prasad, Larissa Grigoryan, Sylvia J. Hysong, Jennifer R. Kramer, Suja S. Rajan, Nancy J. Petersen, Tracey Rosen, Dimitri Drekonja, Christopher Graber, Payal Patel, Paola Lichtenberger, Timothy P. Gauthier, Steven Wiseman, Makoto Jones, Anne Sales, Sarah Krein, Aanand Naik

**Affiliations:** 1 Center for Innovations in Quality, Effectiveness, and Safety (IQuESt) (152), Michael E. DeBakey Veterans Affairs Medical Center, 2002 Holcombe Boulevard, Houston, TX 77030 USA; 2 Rice University, Houston, USA; 3 Department of Family and Community Medicine, Baylor College of Medicine, 3701 Kirby Drive, Suite 600, Houston, TX 77098 USA; 4 Department of Management, Policy and Community Heath, University of Texas (UT) – School of Public Health (SPH), E-319, 1200 Pressler Street, Houston, TX 77030 USA; 5 Infectious Diseases (111F), Minneapolis VA Medical Center, 1 Veterans Drive, Minneapolis, MN 55417 USA; 6 Infectious Diseases Section, VA Greater Los Angeles Healthcare System, David Geffen School of Medicine at UCLA, 11301 Wilshire Blvd, 111-F, Los Angeles, CA 90073 USA; 7 Division of Infectious Diseases, III-i, University of Michigan, 2215 Fuller Road, Ann Arbor, MI 48105 USA; 8 Bruce W. Carter VAMC, 1201 NW 16th Street, Miami, FL 33125-1693 USA; 9 George E. Wahlen Veterans Affairs Medical Center, Mailstop 182, 500 Foothill Drive, Salt Lake City, UT 84148 USA; 10 Department of Learning Health Sciences, University of Michigan Medical School, 209 Victor Vaughan Building, 2054, 1111 E. Catherine St, Ann Arbor, MI 48109-2054 USA; 11 VA Ann Arbor Center for Clinical Management Research, North Campus Research Complex, Building 16-333W, 2800 Plymouth Rd, Ann Arbor, MI 48109-2800 USA; 12 Baylor College of Medicine in Houston, Houston, TX USA

**Keywords:** Antibiotic stewardship, Asymptomatic bacteriuria, Audit and feedback, Guidelines implementation, Dissemination, Urinary tract infection

## Abstract

**Background:**

Antimicrobial stewardship to combat the spread of antibiotic-resistant bacteria has become a national priority. This project focuses on reducing inappropriate use of antimicrobials for asymptomatic bacteriuria (ASB), a very common condition that leads to antimicrobial overuse in acute and long-term care. We previously conducted a successful intervention, entitled “Kicking Catheter Associated Urinary Tract Infection (CAUTI): the No Knee-Jerk Antibiotics Campaign,” to decrease guideline-discordant ordering of urine cultures and antibiotics for ASB. The current objective is to facilitate implementation of a scalable version of the Kicking CAUTI campaign across four geographically diverse Veterans Health Administration facilities while assessing what aspects of an antimicrobial stewardship intervention are essential to success and sustainability.

**Methods:**

This project uses an interrupted time series design with four control sites. The two main intervention tools are (1) an evidence-based algorithm that distills the guidelines into a streamlined clinical pathway and (2) case-based audit and feedback to train clinicians to use the algorithm. Our conceptual framework for the development and implementation of this intervention draws on May’s General Theory of Implementation. The intervention is directed at providers in acute and long-term care, and the goal is to reduce inappropriate screening for and treatment of ASB in all patients and residents, not just those with urinary catheters. The start-up for each facility consists of centrally-led phone calls with local site champions and baseline surveys. Case-based audit and feedback will begin at a given site after the start-up period and continue for 12 months, followed by a sustainability assessment. In addition to the clinical outcomes, we will explore the relationship between the dose of the intervention and clinical outcomes.

**Discussion:**

This project moves from a proof-of-concept effectiveness study to implementation involving significantly more sites, and uses the General Theory of Implementation to embed the intervention into normal processes of care with usual care providers. Aspects of implementation that will be explored include dissemination, internal and external facilitation, and organizational partnerships. “Less is More” is the natural next step from our prior successful Kicking CAUTI intervention, and has the potential to improve patient care while advancing the science of implementation.

## Background

One of the most common reasons for overuse of antibiotics in both acute and long-term care is inappropriate treatment of asymptomatic bacteriuria (ASB), or bacteria in the urinary tract without related urinary symptoms [1–3]. Unnecessary antibiotics given to treat ASB can cause harm in terms of antibiotic resistance, adverse drug effects, and needless expense [4]. The need in the Veterans Health Administration (VHA) to improve this practice is particularly acute, as the VA Antimicrobial Stewardship Task Force reported in April 2016 that 72% of cases of ASB were treated unnecessarily with antibiotics [5].

**Fig. 1:**
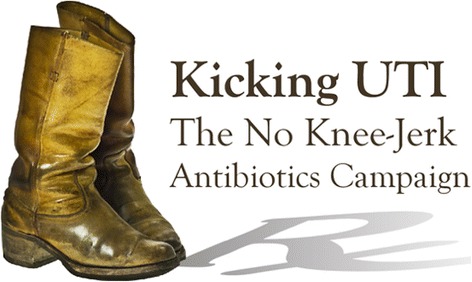
Study logo for Less is More project

We designed and validated a successful antimicrobial stewardship intervention to decrease guideline-discordant management of ASB in Veterans in hospitals and long-term care [6]. This “Kicking CAUTI Campaign” led to a 71% reduction in screening for ASB and a 75% reduction in treatment of ASB at one major VA medical center [7]. The current dissemination project, entitled “Less is More,” will evaluate the effectiveness of the Kicking CAUTI Campaign across four different VHA medical centers while assessing the adoption, fidelity, generalizability, and necessary dose of the intervention (Fig. 1). We will also measure effectiveness of the program with respect to reducing inappropriate screening for and treatment of ASB. Unlike the original Kicking CAUTI intervention, we will include all cases of bacteriuria, occurring with or without a urinary catheter, to be more broadly applicable, as UTI is a much more common reason for antibiotic use than CAUTI.

The Kicking CAUTI Campaign was controlled, grounded in evidence, and employed audit and feedback as part of a multifaceted strategy in acute and long-term care wards [6, 7]. The two main tools used in this intervention are (1) an evidence-based, actionable (“fast and frugal”) algorithm [8, 9] which distills the guidelines into a streamlined clinical pathway and encourages a mindful pause and (2) case-based audit and feedback to train clinicians to use the algorithm within context. The mindfulness engendered by the algorithm then becomes embedded into the mental rules of thumb (heuristics) clinicians use in routine care, thus correcting misleading cognitive biases [9–11]. We chose audit and feedback as part of our intervention, as it had been shown to be successful for reducing overuse of antibiotics in prior studies, [12–17] and it is one of the evidence-based strategies recommended by guidelines on antimicrobial stewardship [18].

Current evidence to support antibiotic stewardship interventions is limited. Although multiple studies suggest that antimicrobial stewardship programs may be associated with improved antibiotic prescribing practices, the quality of the evidence is low, generalizability is unclear, and cause and effect are unproven. For example, in 2016, the Infectious Diseases Society of America issued guidelines on “Implementing an Antibiotic Stewardship Program,” and of the 28 recommendations made, 23 are supported by weak evidence or expert opinion [19]. The Kicking CAUTI intervention represents an advance beyond prior stewardship studies while addressing important knowledge gaps.

**Fig. 2:**
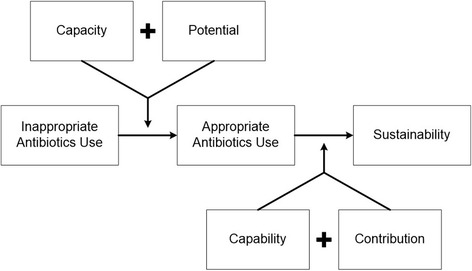
Conceptual Model for the Less is More Intervention

The Less is More project will provide generalizable information for the field of implementation science because a theoretical model guides both the work and the assessment processes. Each aspect of our intervention is linked to a critical concept in the General Theory of Implementation (Fig. 2) [20]. We will measure the “dose” delivered of various components of the intervention and relate the dose intensity to the clinical outcomes. We are also interested in assessing the context in which the intervention occurs, including both individual and institutional factors, using measures that will assist in pooling data across studies. Thus, this project will further contribute to our understanding of the use and utility of key implementation strategies, including external and internal facilitation [21, 22] and audit and feedback, in both inpatient and long-term care settings.

## Methods and design

### Study design

The study uses interrupted time series design with four contemporary control sites to test an intervention using audit and feedback of healthcare providers to improve their compliance with ASB guidelines. The intervention will be conducted at four geographically diverse VA facilities, with external facilitation provided by the coordinating site in Houston. The control sites will be four VA facilities in the same geographic area as each intervention site to account for any potential regional differences in policies or practices regarding antibiotic stewardship.

### Specific aims and hypotheses

*Specific Aim 1*, which will be performed in year 1, is to assess context, barriers, and facilitators at each site prior to implementation. This work includes baseline measurements of ASB diagnosis and treatment to inform intervention implementation.

*Specific Aim 2*, performed over years 1–3, is to evaluate implementation of a scalable version of the Kicking CAUTI intervention (individualized, case-based audit and feedback) in four geographically distinct VA medical centers, including both acute and long-term care settings, with four contemporaneous controls. We hypothesize that the intervention observation periods will exhibit a decrease in screening urine cultures ordered (primary outcome), decreased use of antimicrobials, and a decreased number of episodes of *Clostridium difficile* infection, without an increase in urinary source bacteremia, among intervention sites compared to control sites (Aim 2A). We also hypothesize that higher levels of adoption and fidelity will be associated with better clinical outcomes (Aim 2B).

*Specific Aim 3*, performed throughout the 3 years of the project, is to assess the financial implications of the intervention through a budget impact analysis [23].

### Conceptual model

Our conceptual framework for the development and implementation of a scalable version of the Kicking CAUTI intervention draws on May’s proposed General Theory of Implementation, which links Normalization Process Theory (NPT), [20, 24] with other sociological and psychological theories (Fig. 2). The General Theory of Implementation describes constructs that must be assessed and that serve as potential targets in the process of implementing a complex intervention successfully [20]. These include factors that improve the *capacity* of agents to cooperate and coordinate in order to implement (e.g., material resources, social roles and norms, and cognitive resources), as well as their *potential* (individual intention and collective commitment) for implementation. A complex intervention succeeds and becomes embedded in daily routines through the *capability* of individuals to operationalize a complex intervention. Finally, a sustainable intervention requires ongoing *contribution* by the parties involved. We expect that over time, the mindful pause prompted by our algorithm will become a standard heuristic through which guideline-concordant behaviors will be normalized into routine care. Our study measurements and variables are linked to our conceptual model (Table 1).

**Table 1:** Variables Organized by Project Aims and Conceptual Model

Variable or data elements	Source	Conceptual mapping and purpose
Aim 1: Pre-Implementation Assessment of Intervention Sites		
Identification of local champions	Site elements questionnaire (Appendix B)	Capacity: Social roles
Identification of critical site elements		Capacity: Material resources
		Potential: Collective contribution
Daily workflow around bacteriuria	Process mapping	Capacity: Social roles and material resources
Perception of organizational readiness to change	ORCA (Appendix C)	Potential: Collective commitment
Perception of leadership support of change effort		Capacity: Social norms
Social norms	Kicking CAUTI Survey (Appendix D)	Capacity: Social norms
Self-efficacy		Potential: Individual intention
Outcome expectancy		Potential: Individual intention
Guidelines familiarity and acceptance		Capacity: Cognitive resources
Behavior (self-reported)		Capacity: Cognitive resources
Aim 2: Implement the Kicking CAUTI Intervention		
Aim 2A: Measure effectiveness of the intervention in terms of clinical outcomes (intervention and control sites)		
Number of urine cultures ordered	CDW	Clinical outcomes reflect contribution: sense making, cognitive participation, collective action, and reflexive monitoring
Number of antimicrobial starts	CDW	
Number of antimicrobial starts after urine culture	CDW	
Number of episodes of *C. difficile* associated disease	CDW	
Number of urinary source bacteremias	CDW	
Number of episodes of UTI and ASB	Chart review^a^	
Percentage of episodes of ASB treated with antimicrobials	Chart review^a^	
Percentage of episodes of UTI not treated	Chart review^a^	
Aim 2B: Measure the adoption and fidelity of the intervention process (intervention sites)		
Number of cases presented as audit and feedback sessions/total cases per month	Recorded by site champion (adoption)	Capability: Workability
Number of staff who participate in case presentations/total potential participants per month	Recorded by site champion (adoption)	
Number of surveys completed/distributed	Manual count (adoption)	
Method of delivery of case-based audit and feedback	Recorded by site champion (fidelity)	
Leadership awareness of intervention	% emailed facility reports opened (adoption)	Capability: Integration Contribution: Collective action Contribution: Reflexive monitoring
Algorithm penetration	Number of pocket cards distributed (adoption)	
Integration of intervention into existing antimicrobial stewardship program	Annual report includes Kicking CAUTI program or elements (adoption)	
Aim 3: Conduct a Budget Impact Analysis (Intervention sites)		
Clinical utilization costs (pre, during, post)	Time logs, surveys, CDW, HERC, MCAS, literature	Capability: Workability Contribution: Coherence or sense-making
Intervention costs		

^a^Individual chart review will be triggered by positive urine cultures

Abbreviations: *ORCA* organizational readiness to change, *CDW* corporate data warehouse, *HERC* Health Economics Resource Center, *MCAS* Managerial Cost Accounting System

### Overview of intervention design and timeline

**Fig. 3:**
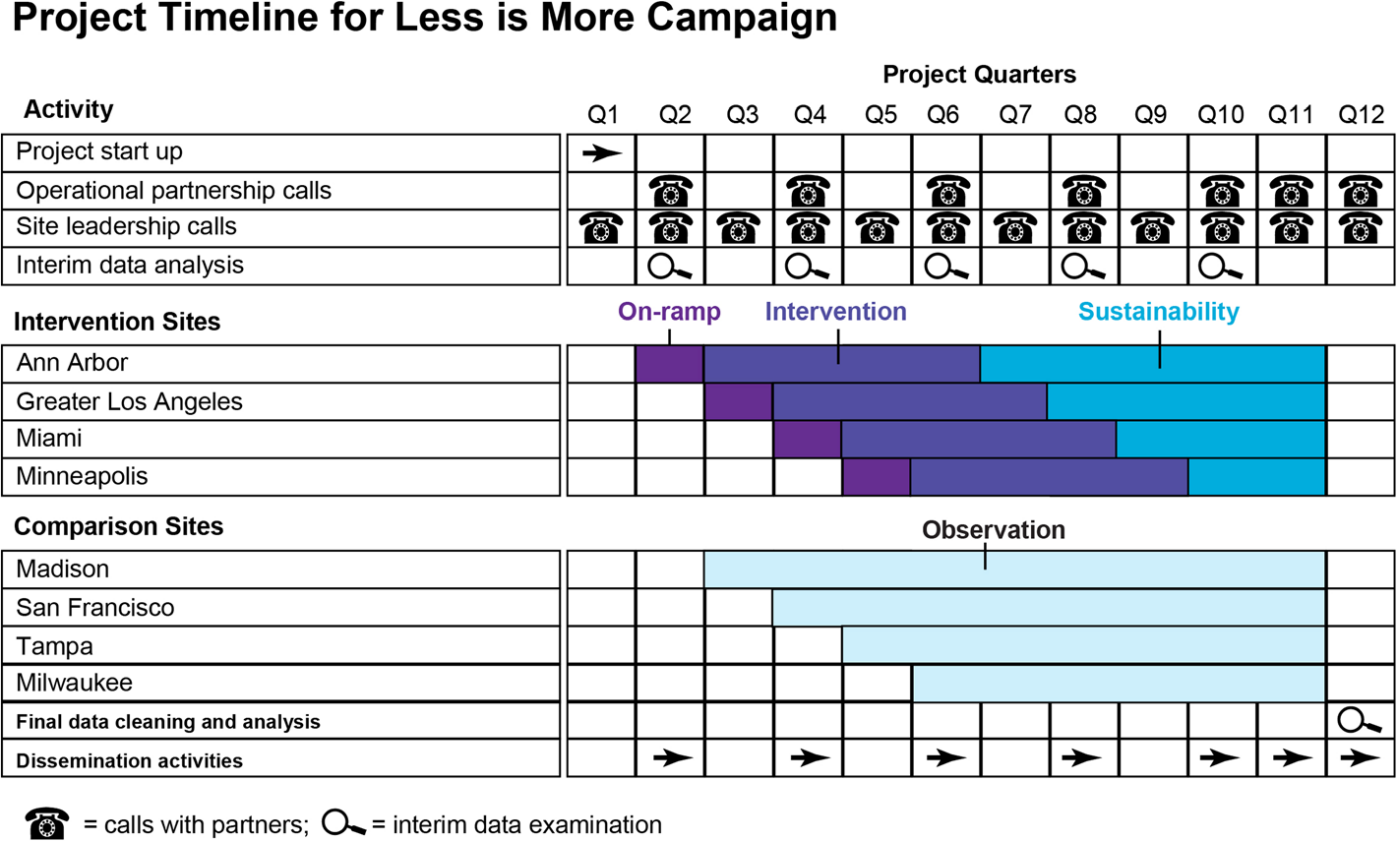
Project Timeline for Roll-out of the Less is More Intervention

Our overall objective in this proposal is to evaluate the effectiveness and implementation of a streamlined, scalable version of the Kicking CAUTI Campaign across four diverse VA medical centers. To address scalability, we will gather our primary clinical outcome data from the VA Corporate Data Warehouse (CDW), which is a relational database extracted directly from patients’ electronic medical records containing all laboratory results, including cultures. We will train existing antimicrobial stewardship champions at each site (generally infectious diseases physicians and infectious diseases pharmacists) to deliver the intervention, thus using existing resources. The 3-year project allows time for three phases at each site: project *startup* (surveys and baseline data measurements), 12 months for *active intervention* implementation, and a *sustainability phase* during which active support from the centralized coordinating site ceases (Fig. 3). Audit and feedback will take place on two levels for this project. Provider level audit and feedback will consist of case based training in applying the ASB guidelines to individual patients. The local champions will deliver this in small group settings. Facility level audit and feedback will consist of information on urine cultures ordered and antibiotic use on a monthly basis, compared to other participating facilities (intervention and comparison). The facility level feedback will be given to each site champion for distribution to relevant parties within their site.

### Study setting

We will conduct this study in inpatient acute medicine units and long-term care units in the following four major VA medical centers: Ann Arbor, Greater Los Angeles, Miami, and Minneapolis. Each of these sites has both acute care and community living centers. These sites were chosen because each has a motivated local physician champion who is early to mid-career and has the bandwidth to commit to this project, with support from a senior antimicrobial stewardship mentor on site. The proposed comparison sites are Madison, San Francisco, Tampa, and Milwaukee. The four comparison facilities have been chosen from the same geographic areas as the intervention sites and matched in terms of teaching status, characteristics of their current antimicrobial stewardship program, ward types, facility level, ICU level, and number of beds.

### Participants

The goal of this project is to teach providers to distinguish between UTI and ASB and to make the appropriate choice to withhold antimicrobials when the patient has ASB. Thus, the health care providers who order urine cultures and antimicrobial agents are the subjects of the intervention itself, and their involvement will be to receive education about the relevant guidelines and audit and feedback about their management of bacteriuria, both catheter-associated and not catheter-associated. The clinical outcomes for this project, such as number of urine cultures ordered and antibiotic use for bacteriuria, will be monitored in the Veterans who are inpatients in acute care facilities or residents of long-term care facilities within intervention and control sites. The local project team at each implementation site consists of a site champion (an infectious diseases physician), an infectious diseases pharmacist, and a research assistant, plus any other members recruited by the site champion.

### Materials

#### Surveys

We will administer three different surveys in year 1, each for a different purpose. These include the site elements questionnaire, [25] the context scale of the Organizational Readiness to Change Assessment (ORCA) instrument, [26] and the Kicking UTI survey [27]. The ORCA was initially developed through the Ischemic Heart Disease Quality Enhancement Research Initiative (QUERI) program and has been used in several QUERI related studies [26, 28, 29]. We plan to administer the ORCA context assessment, which asks about leadership culture, staff culture, leadership practice, and evaluation/accountability. The main purpose of the Kicking CAUTI survey is to use this previously validated tool to assess baseline awareness of the CAUTI and ASB guidelines and to identify the cognitive biases driving overtreatment of ASB, which will enable us to target our intervention teaching materials. The Kicking UTI survey will be administered widely to licensed providers who order antibiotics, nurses, and clinical nurse assistants. The site elements questionnaire [25] will ask questions about available resources, such as whether any antimicrobial restriction policies are in place, whether the site has an infectious diseases physician, etc. A single site elements survey will be completed per site, by the site champion, about local resources available for antibiotic stewardship.

### Algorithm and teaching materials

The main tool used in our intervention is our evidence-based, actionable (‘fast and frugal’) Kicking UTI algorithm [8, 9]. This algorithm distills the guidelines into a streamlined clinical pathway and encourages a mindful pause. Specifically, the algorithm applies to any patient who is being assessed for possible UTI. The provider using the algorithm is instructed to ask themselves two questions before reflexively ordering a urine culture or starting empiric antibiotics. These questions are: “Does the patient have any of these evidence-based symptoms of UTI,” and “If yes, then could a non-urinary condition account for these symptoms?” During the intervention, the project team will create teaching materials based on specific cases, each built around this algorithm. Delivery of these case-based teaching materials to the providers involved in the case constitutes our audit and feedback. Each case is presented in a brief PowerPoint which begins with the algorithm, hyperlinked at each decision point. The provider receiving the feedback can thus explore possible choices and options during the brief educational session delivered by a member of the local project team (physician, pharmacist, or research assistant).

### Intervention

Our intervention uses case-based audit and feedback to train clinicians to use the algorithm within context. The mindfulness engendered by the algorithm then becomes embedded into the mental rules of thumb. We chose audit and feedback as part of our intervention, as it had been shown to be successful for reducing overuse of antibiotics in prior studies [12–17], and it is one of the evidence-based strategies recommended by guidelines on antimicrobial stewardship [18].

The local research assistant, supported by the Houston research coordinator, will gather episodes of positive urine cultures and classify the cases as ASB or UTI. The list of cases and their outcomes (ASB or UTI, treated appropriate or inappropriately) will be placed on each site’s share drive on a weekly basis. The local pharmacy champion will deliver feedback on cases in real time (shortly after antibiotics are prescribed) through post-prescription antimicrobial review, using the algorithm, and typically by telephone. The Houston-based team will also create appropriate teaching materials for each site’s specific cases and place these on the shared drive, on a weekly basis. These case-based teaching materials will take the form of brief (10 min or less) PowerPoint presentations relevant to the cases that occurred in their site during the past week. The physician champion and research assistant will deliver audit and feedback via teaching cases in small group settings, such as resident teaching conferences, infectious diseases rounds with inpatient medicine teams, and hospitalist meetings. The goal is to deliver the audit and feedback to the relevant providers within a week of the positive urine culture report. The local champion will organize in services with the long-term care staff to present cases from long-term care. The research assistant will also receive pocket cards with the Kicking UTI algorithm for wide distribution within the local setting. Targets for audit and feedback delivery will be set, such as one case per week in acute care, two in-services per month in long-term care, and one teaching conference per quarter with residents and hospitalists.

### Economic evaluation

The economic evaluation (Aim 3) will assess the financial implications of the intervention through a budget impact analysis (BIA). The BIA will be performed from the VHA’s perspective using the best practice guidelines outlined by Sullivan et al. [30]. The BIA will estimate the financial impact per quarter on the VHA’s budget to implement and sustain the intervention. The analysis will help the VHA and future VHA sites implementing the intervention to be financially prepared before initiating the intervention and will also establish the business case for the intervention. The intervention primarily aims to reduce clinical utilization as defined by the number of screening urine cultures ordered, unnecessary treatment of ASB, use of antimicrobials, and number of episodes of *Clostridium difficile* infection, without an increase in urinary source bacteremia. The BIA aims to capture the resource and cost changes associated with these reductions/changes, and also capture the cost of the intervention itself.

### Analysis

#### Analysis plan for aim 1

Descriptive and summary statistics will be calculated using data from the surveys performed in Aim 1. For the Kicking CAUTI survey, we will calculate the knowledge score and will use identified gaps in knowledge to tailor audit and feedback materials. Guideline familiarity, social norms, outcome expectancy, self-reported behavior, and self-efficacy will be compared between sites and by characteristics such as type of facility. The other two surveys (ORCA and the site elements survey) will provide information relevant to local tailoring of the intervention.

#### Analysis plan for Aim 2A

The purpose of Aim 2 is to measure the effectiveness of the intervention in changing clinical outcomes of interest, in comparison to four control sites.

##### Variables for Aim 2A

We will use data from the CDW to measure the effectiveness of the intervention in changing clinical outcomes of interest, in comparison to four control sites. The number of urine cultures at each site is the primary outcome, while secondary outcomes include the number of antibiotic courses started, number of antibiotic courses started within 48 h of a urine culture, and number of episodes of *C. difficile* infection. We will obtain the number of episodes of urinary source bacteremia (urosepsis) using a previously published definition (urine culture and blood culture have same organism and urine culture was collected within 0–7 days prior to the blood culture) [31]. We will also obtain the number of patient days, or patient bed-days of care, to provide a denominator for standardization across facilities. The primary outcome is the total number of urine cultures ordered because ordering a urine culture is the first step that leads to overtreatment of ASB, and a positive urine culture is a powerful (but often incorrect) stimulus for use of antibiotics [32].

An additional set of measures will be obtained in both intervention and control sites by selecting patients with positive urine cultures from each site for chart review. Capturing whether a urinary catheter was present, whether each positive urine culture represented UTI, CAUTI or ASB, and whether antibiotics were given to treat the urine organisms will require chart review by a research assistant using our previously validated and standardized methods of chart review [9, 33]. We expect high numbers of positive urine cultures from each site, and it will not be feasible or necessary to perform chart reviews for all positive cultures. We will randomly select patients with positive cultures from each site on a daily basis, varying the time of collection.

##### Analysis for Aim 2A

We will use interrupted time series with segmented regression analysis (ITS/SRA) [34, 35]. Segmented regression analysis will be used to evaluate the effectiveness of the intervention in changing clinical outcomes of interest, in comparison to four control sites. We will conduct separate segmented regression analyses for the intervention and control sites. Data for each outcome variable will be aggregated monthly, and the time period will be divided into before, during, and after intervention segments, with separate intercepts and slopes estimated in each segment. Comparing the effect in the intervention sites with that in the control sites will allow separating the intervention effect from underlying secular trends [34, 36].

##### Power for Aim 2A

Our study is powered around our primary outcome of interest, the number of urine cultures ordered (inappropriate screening for ASB). Based on our preliminary data, we expect intervention sites to provide approximately 900 urine cultures per month prior to the intervention, and 250 urine cultures per month after all sites are participating in the intervention. Because we will use existing data in CDW, we will be able to obtain our outcomes for 12 months pre-intervention (baseline), 12 months during the intervention, and 6–12 months post-intervention (sustainability phase) (Fig. 3). Our 36 monthly time points will provide adequate power to detect significant trends at different periods [37]. Segmented regression analysis will fit a model with three segments, corresponding to the pre-intervention, intervention, and post-intervention periods.

#### Analysis plan for Aim 2B

The purpose of Aim 2B is to assess whether the adoption and fidelity (i.e. completeness of implementation) and the dose of the intervention are related to clinical outcomes (the number of urine cultures ordered).

##### Variables for Aim 2B

The two variables of most interest used to measure “completeness” of implementation are the number of cases in which feedback was delivered out of the total number of cases of positive urine cultures and the number of participants in intervention activities out of eligible participants.

##### Analysis for Aim 2B

The analysis for Aim 2B will assess whether more complete implementation is associated with better clinical outcomes (specifically the number of urine cultures ordered, standardized by bed-days). Data will be tabulated monthly for each VA facility.

Because facilities will have varying numbers of patients and urine cultures and because the outcome measures may have non-normal distributions, a preferred method of analysis involves the use of generalized linear mixed models (GLMM) or generalized estimating equations (GEE) [38]. An analysis such as GEE will allow monthly data to be nested within a facility. The regression models will include the month, facility, facility size, facility type, context (ORCA), patient characteristics, nurse staffing levels, antibiotic stewardship FTE, and the process measures representing the completeness of the implementation. To determine the subset of process measures most associated with the clinical outcome to use in the multivariable regression, we will initially conduct univariate analyses of each measure with the outcome. This includes correlation of continuous measures such as percentage of total cases per month that are presented as audit and feedback with the number of urine cultures ordered (standardized by bed-days). Analysis of variance will be used for assessing the association of categorical process measures, such as method of audit and feedback delivery (such as in person or by telephone), with the clinical outcome. Those process measures which are significant in the univariate analysis will be included in the GEE analysis. In the multivariable regression, the parameter estimates for process measures will indicate how much a change in the process measure will impact the clinical outcome. A negative parameter estimate will indicate that as the “completeness” of that implementation process measure increases, the clinical outcome (urine cultures) will decrease, after accounting for differences in variables such as facility size, ORCA, facility type (teaching or non-teaching), etc.

##### Power for Aim 2B

We will have 12 monthly measurements of the clinical outcomes during the intervention year for each of the four facilities, hence a total of 48 measures. This sample size will allow the impact assessment of approximately five factors such as the facility type and the process measures on the clinical outcome, based on the general rule-of-thumb proposed by Cornfield of 10 observations for each independent variable in a linear regression model [39].

#### Analysis plan for Aim 3

The BIA will estimate costs for (1) the pre-intervention time period, in order to obtain the baseline utilization based on current practices, which contribute to clinical utilization costs of urine cultures ordered, use of antimicrobials, number of episodes of *C. difficile* infection, and urinary source bacteremia in the four VA medical centers before the intervention is put in place; (2) the intervention time period, in order to obtain clinical utilization costs during the intervention, in addition to the costs associated with the intervention implementation (including barrier assessment for initiating the intervention, intervention development, training and delivery); and (3) the post-intervention/sustainability time period, in order to capture the clinical utilization costs and other costs associated with sustaining the intervention i.e. training, delivery, and supervision involved with sustaining the intervention. Static modeling will be used for the BIA [40]. The patient population of interest is all inpatients on acute medical or long-term care wards at the intervention sites. The unit of analysis is a positive urine culture. The same patient may have more than one urine culture; we count these as separate episodes if more than 7 days apart. These recurrent episodes will be included in the BIA in order to capture the actual episode volume and costs for each VHA medical center. Inflation adjustment and discounting will not be applied because dollar values will be standardized to those from the final fiscal year of the study. Cost derived for the Kicking UTI intervention, Managerial Cost Accounting System (MCAS), and other clinical utilization costs will be adjusted for geographic variations. Cost derived from the standardized Health Economics Resource Center (HERC) data will not require geographic adjustments.

### Ethical approval

This study protocol has been approved by the institutional review board at Baylor College of Medicine and the Research & Development Committee at the Michael E. DeBakey Veterans Affairs Medical Center, as the coordinating center. Each intervention site also obtained local IRB approval.

## Discussion

The intervention we propose appears straightforward—convince providers to stop using antibiotics in asymptomatic patients when the antibiotics are unnecessary, potentially harmful, and costly. However, doing this practical work and doing it well requires changing physicians’ and other health care providers’ deeply held paradigms about the risk of bacteriuria (overestimated) and the risks of antibiotic use (underestimated) [41]. Historically, the healthy bladder was thought to be a sterile site, an assumption now proven incorrect by modern genomic sequencing, which demonstrates that the healthy bladder has robust bacterial and viral life within [42, 43]. Another barrier to the successful implementation of our stewardship intervention is the complexity of the practice guidelines themselves [44, 45] and the difficulty of applying 51 pages of evidence to an individual patient at the point of care. Additionally, the perceived needs of the individual patient in a moment of diagnostic uncertainty tend to outweigh concerns for theoretical future harms caused by damage to a patient’s microbiome and selection for resistant organisms. The perceived needs of individual patients under care also tend to outweigh societal needs for effective antibiotics in the future.

We propose to counter these challenges with a solution that is relatively simple. We started with 51 pages of guidelines and created a multistep algorithm, with input from the guidelines’ authors. We went through iterative revisions of the multistep algorithm with targeted end-users, and we distilled it into a two-step process that corrects cognitive biases and empowers the provider to withhold urine testing and treatment [9]. This algorithm is the focus of our audit and feedback activities. These simplifications were developed using the Evidence Integration Triangle framework, which emphasizes the need to keep interventions simple, participatory, and practical to promote rapid adoption of clinical practice guidelines [46].

Moving from our prior two-site study to a four-site intervention with four control sites might seem incremental, but this scale-up is an important shift in the translation pipeline. Our initial “Kicking CAUTI” study was in the T2 phase of knowledge dissemination—applying evidence based guidelines to improve clinical practice. Now with “Less is More” we move into T3 translation, because we are moving guidelines into widespread health practice through dissemination research [47, 48]. We are also making a leap moving from a single, trusted local champion who originated the project to a more sophisticated model of internal and external facilitation. The original Kicking CAUTI campaign was successful in large part through the energy, involvement, and reputation of the trusted local leader. In order to disseminate beyond the reach of a single individual, yet maintain the role of the trusted local leader, we propose an internal/external facilitation model [21]. The Houston team will provide external facilitation in the form of subject matter expertise, teaching materials, and troubleshooting implementation barriers, while the local leaders, each well-known and charged with antibiotic stewardship in their home facility, will provide internal facilitation of the stewardship intervention [49].

Multiple organizational partners have an interest in helping our work succeed and planning for wide-scale dissemination. These partners, who will be involved in reviewing results and providing project direction on a twice yearly basis, include the VA National Center for Patient Safety, the VA Antimicrobial Stewardship Task Force, the VA National Infectious Diseases Service, and the Centers for Disease Control program “Get Smart for Healthcare.” We also have a Veteran representative on the team so that we can have a bi-directional discussion with Veterans’ associations about our work and how it can address Veterans’ healthcare priorities.

In summary, “Less is More” moves from a proof-of-concept effectiveness study to an implementation study involving significantly more sites, and uses the May’s General Theory of Implementation to embed the intervention into normal processes of care with usual care providers. “Less is More” is the natural next step from our prior successful Kicking CAUTI intervention, with a more sophisticated approach to behavioral science and to evaluation of implementation.
